# Avidity-Dependent Programming of Autoreactive T Cells in T1D

**DOI:** 10.1371/journal.pone.0098074

**Published:** 2014-05-20

**Authors:** Ivana Durinovic-Belló, Vivian H. Gersuk, Chester Ni, Rebecca Wu, Jerill Thorpe, Nicholas Jospe, Srinath Sanda, Carla J. Greenbaum, Gerald T. Nepom

**Affiliations:** 1 Benaroya Research Institute at Virginia Mason, Seattle, Washington, United States of America; 2 University of Rochester School of Medicine, Rochester, New York, United States of America; 3 University of Washington School of Medicine, Seattle, Washington, United States of America; University of Michigan Medical School, United States of America

## Abstract

Fate determination for autoreactive T cells relies on a series of avidity-dependent interactions during T cell selection, represented by two general types of signals, one based on antigen expression and density during T cell development, and one based on genes that interpret the avidity of TCR interaction to guide developmental outcome. We used proinsulin-specific HLA class II tetramers to purify and determine transcriptional signatures for autoreactive T cells under differential selection in type 1 diabetes (T1D), in which insulin *(INS)* genotypes consist of protective and susceptible alleles that regulate the level of proinsulin expression in the thymus. Upregulation of steroid nuclear receptor family 4A (NR4A) and early growth response family genes in proinsulin-specific T cells was observed in individuals with susceptible *INS*-VNTR genotypes, suggesting a mechanism for avidity-dependent fate determination of the T cell repertoire in T1D. The NR4A genes act as translators of TCR signal strength that guide central and peripheral T cell fate decisions through transcriptional modification. We propose that maintenance of an NR4A-guided program in low avidity autoreactive T cells in T1D reflects their prior developmental experience influenced by proinsulin expression, identifying a pathway permissive for autoimmunity.

## Introduction

Insulin is an important autoantigen in type 1 diabetes (T1D) autoimmunity, most likely the primary causative antigen in the spontaneously diabetic NOD mouse, and a target autoantigen in Caucasian individuals with the highest HLA-DRB1*04, DQ8 risk-haplotype [1]–[3]. T cell reactivity to a major proinsulin epitope 73–90 occurs as a marker of autoreactivity in pre-diabetic DRB1*04:01 individuals and during the course of disease progression, when extensive intra- and inter-molecular spreading of T cell responses extends the response to multiple islet-associated targets [4]. Polymorphisms at the proinsulin locus correlate with genetic susceptibility and with the frequency of proinsulin-specific T cells in the periphery; specifically, the insulin (INS) variable number tandem repeat (VNTR) class III (141–209 repeats of a 14-base pair-long consensus motif) correlates with a 3-4-fold higher expression level for proinsulin in the thymus and is associated with protection from T1D, whereas INS VNTR class I alleles (26–63 repeats) in homozygous form are associated with lower levels of INS expression in thymus, higher levels in beta cells, and an increased relative risk for T1D that ranges between 1.9–5 [5], [6]. We have previously shown that the frequency of proinsulin-specific CD4 T cells in peripheral blood of T1D subjects and healthy controls is higher in DRB1*04:01 subjects homozygous for INS VNTR class I as compared with DRB1*04:01 T1D and healthy controls with INS VNTR class III [7], consistent with a mechanism for the protective effect of the class III alleles, involving more efficient deletion of INS-autoreactive T cells, a process known to be antigen dose dependent [8], [9].

Thresholds for antigen-dependent negative selection in the thymus are under complex regulatory control, including a set of transcription factors that integrate the strength of antigen recognition with subsequent T cell fate determinism. The nuclear receptor family 4A (NR4A) family of genes is integral to this function and has been directly implicated in murine models of avidity-dependent autoimmunity, in which NR4A gene expression is proportional to the strength of T cell receptor (TCR) signaling [10], [11]. Using HLA class II tetramers for proinsulin epitopes presented by HLA-DR4 molecules, we purified antigen-specific autoreactive CD4 T cells from both control and T1D HLA-DRB1*04:01 subjects with different INS VNTR genotypes, and compared transcriptional profiles for NR4A and related gene families. T cells from both control and T1D subjects with the T1D-susceptible, INS VNTR I genotype expressed high levels of NR4A family members, linking disease susceptibility with a key homeostatic pathway for T cell regulation. This effect was most pronounced in a subpopulation of T cells with intermediate avidity for the proinsulin epitope, positioning this mechanism at a stage distinct from negative selection of high avidity cells.

## Results

### Detection of proinsulin-specific CD4 T cells

**Figure 1:**
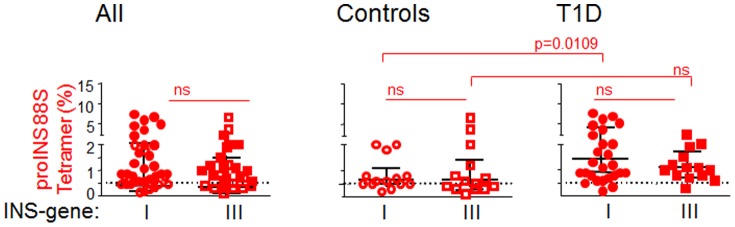
Influence of INS genotype on the frequency of proinsulin-specific CD4+ T cells. These results are shown for all subjects (left panel), healthy control subjects (middle panel), and T1D subjects (right panel), matched for HLA-DRB1*04:01 and tested using the proinsulin 88S tetramer. Differences in the distribution of tetramer positivity among the groups were calculated using Mann-Whitney U test; an earlier study including some of these subjects has been reported (7). Each symbol represents one tested subject. Black horizontal lines represent medians and inter-quartile ranges. Dashed horizontal lines indicate threshold binding of the negative-control tetramer. Susceptible INS gene I  =  INS VNTR I/I; Protective INS gene III  =  INS VNTR I/III or III/III.

HLA class II tetramers have been used for more than a decade to identify antigen-specific CD4 T cells in peripheral blood for a wide variety of epitopes, including analyses in human autoimmune disease [12]. These studies have shown that CD4 T cells specific for proinsulin, glutamic acid decarboxylase, and IA2 are present at low frequency in both healthy and T1D subjects, with the latter expressing characteristics consistent with prior in vivo activation [13]. A prevalent epitope specificity identified in T1D in HLA-DRB1*04:01 subjects is the peptide-MHC complex consisting of proinsulin 76–90 bound by HLA-DR4 molecules, documented by functional and tetramer-binding analyses [1], [14]. In the latter, modification of proinsulin peptide 76–90 by a K→S substitution at MHC anchor position 9 (proinsulin 88S) stabilizes the pMHC monomer, enabling the use of proinsulin pMHC tetramers that recognize 10–40 proinsulin-specific CD4 T cells per million PBMC [14]. We have previously shown that the frequency of these cells correlates with a polymorphism associated with the proinsulin gene promoter region, an effect attributed to differential levels of proinsulin expression in the thymus [6], [7], [15]. Figure 1 summarizes these data, in which INS genotype and disease status influence the number of proinsulin-specific CD4 T cells recovered from peripheral blood through tetramer-based flow cytometry.

For each of the subjects shown in Figure 1, we sorted tetramer-positive T cells and purified RNA for quantitative PCR analyses, as previously described [7]. A panel of 31 candidate gene transcripts was investigated, based on a prior exploratory test set selected for increased gene expression in antigen-specific CD4 T cells in T1D (Table S1). Using a threshold minimum of 2,500 cells and 2 ng RNA recovered, we were able to generate expression analysis from ten healthy and seven T1D HLA-DRB1*04:01 subjects. A set of three consistent housekeeping genes was included in each analysis, and all expression changes were expressed as a ratio compared with these internal controls.

### Influence of INS genotype on expression profiles

**Figure 2:**
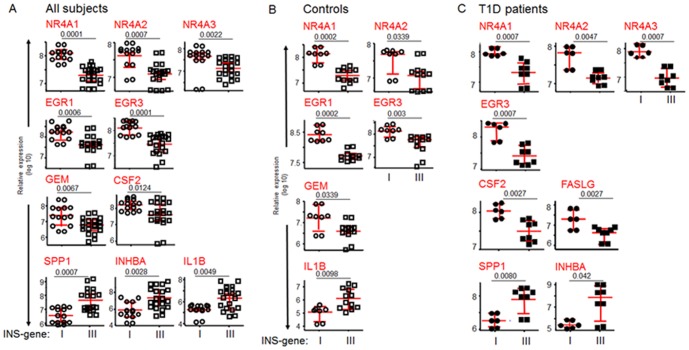
Relative expression panels for all transcripts of CD4 T cells binding the proinsulin 88S tetramer. Expression panels show significant differences between VNTR I and VNTR III subjects for all subjects **(A)**, healthy individuals **(B)**, and T1D patients **(C)**. Symbols represent individual RNA samples tested in duplicate. The differences in the level of transcript expression between INS genotypes were calculated using a Mann-Whitney U test. Longer horizontal lines indicate the medians and small horizontal lines indicate the inter-quartile ranges. Only plots reaching statistically significant difference among the groups are shown. Susceptible INS-gene I  =  INS-VNTR I/I; protective INS-gene III  =  INS-VNTR I/III or III/III.

As shown in Figure 2A, when all subjects analyzed were stratified by INS genotype, ten transcripts present in the antigen-specific CD4 T cells showed significant differences in levels of expression, with seven of those transcripts being increased in the VNTR I (T1D susceptible) group. Notably, all of the NR4A and early growth response (EGR) genes in the panel were elevated in the insulin-specific T cells from VNTR I subjects. A parallel analysis was performed on the same CD4 T cell samples sorted with an HLA-matched tetramer to a hemagglutinin epitope of influenza, and none of the transcripts in Figure 2A showed differences among the influenza-specific cells between subjects with INS VNTR I and III (Figure S1).

When we subdivided these same subjects according to their disease status, three transcripts were overexpressed in both healthy control (Figure 2B) and T1D subjects (Figure 2C) with the INS VNTR I (susceptible) genotype: NR4A1, NR4A2, and EGR3. Two other related gene family members, NR4A3 and EGR1, were also significantly overexpressed in the VNTR I subjects from either the T1D or the control cohort, respectively. These genes displayed a consistent pattern of higher T cell transcripts in subjects with INS VNTR I, the polymorphic variant associated with susceptibility to T1D, and with low levels of thymic insulin expression. Because a similar expression increase was seen in both healthy and T1D subjects, this finding indicates that the higher expression profiles in antigen-specific CD4 T cells were not secondary to disease status, but are consistent with an effect linked to INS genotype for NR4A and EGR gene expression in the antigen-specific T cells.

**Figure 3:**
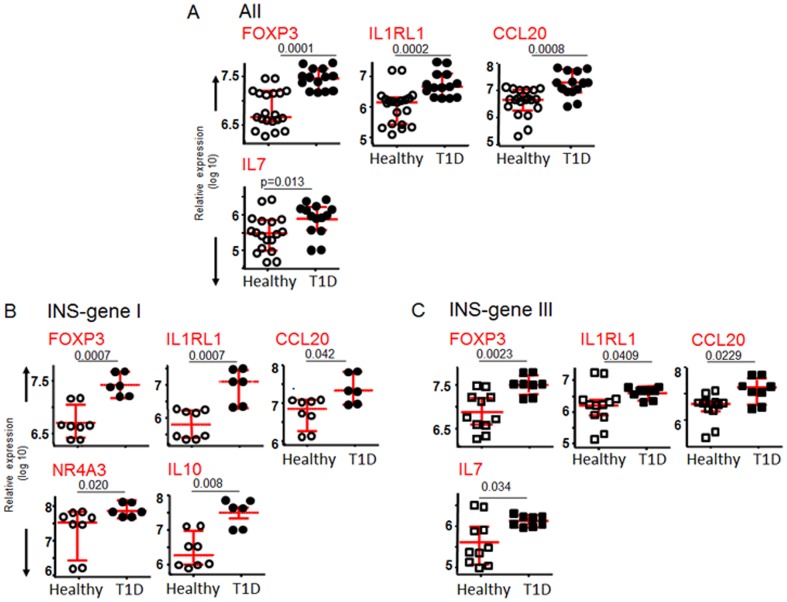
Relative expression plots for significant genes that distinguish between healthy individuals and T1D patients. Patients with both INS genotypes (**A**), those with the VNTR I-susceptible INS genotype (**B**), and VNTR III-protective INS genotype (**C**) are shown. Horizontal lines indicate medians and inter-quartile ranges. The differences in the level of transcript-expression between healthy individuals and T1D patients were calculated using a Mann-Whitney U test. Only plots reaching significant difference between the groups are shown.

A few transcripts were identified that distinguished control from T1D subjects, including activation-associated markers CCL20, IL-1RL1, and Foxp3, all of which were higher in the T1D profiles independent of VNTR genotype (Figure 3). IL-1RL1, a cell surface receptor for IL-33 [16], is a novel candidate for a disease or stress-related T cell marker in T1D.

### Expression profiles as a function of TCR avidity

**Figure 4:**
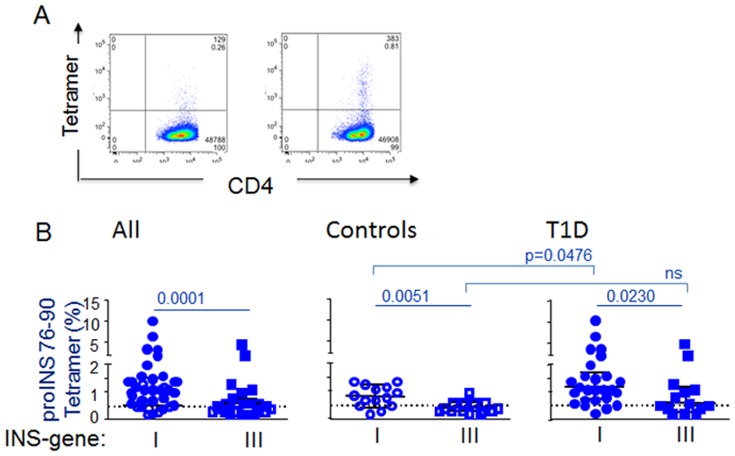
Flow cytometry profile of CD4+ T cells. A T1D patient with the susceptible INS VNTR I/I genotype is shown **(A)**. Staining with the proinsulin 76–90 tetramer (left) and the 88S tetramer (right) is shown. Influence of INS genotype on the frequency of proinsulin-specific CD4+ T cells **(B)**, shown for all subjects (left panel), healthy control subjects (middle panel), and T1D subjects (right panel), matched for HLA-DRB1*04:01 and tested using the proinsulin 79–90 tetramer. Differences in the distribution of tetramer positivity among the groups were calculated using a Mann-Whitney U test; an earlier study including some of these subjects has been reported (7). Solid horizontal lines represent medians and inter-quartile ranges. Dashed horizontal lines indicate threshold binding of the negative control tetramer. Susceptible INS gene I  =  INS VNTR I/I; protective INS gene III  =  INS VNTR I/III or III/III.

**Figure 5:**
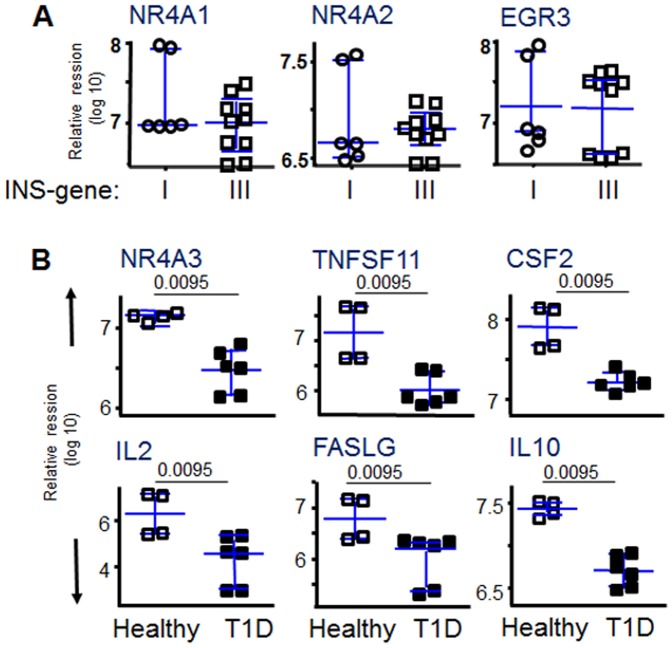
Influence of INS genotype on expression profiles of proinsulin-specific CD4+ T cells. Relative expression profiles are shown for all subjects matched for HLA-DRB1*04:01 and selected for high antigen avidity using the proinsulin 76–90 tetramer **(A)**. Several genes showed significant transcript differences between control and T1D subjects matched for the INS VNTR III diabetes-protective genotype, all p<0.01. **(B)**.

High avidity TCR recognition of self-antigens expressed by thymic epithelial cells results in T cell deletion, through a mechanism known as negative selection [17]. In T1D, this mechanism has been modeled with the use of humanized mice transgenic for HLA and for human TCR specific for islet autoantigens [18]. In order to assess the relationship between avidity selection and the expression profile, we compared T cell transcripts using the modified proinsulin pMHC tetramer described above with a parallel study on the same samples using the native proinsulin epitope 76–90 is bound to HLA-DR4 molecules. In the latter case, the resulting pMHC complex has limited binding restricted to CD4 T cells with high avidity recognition for this epitope [14]. As predicted by the negative selection model, the frequency of these high avidity tetramer-binding cells is very low (Figure 4A). We detected CD4 T cells that bound tetramers with this high avidity phenotype more frequently in subjects with INS VNTR I compared with VNTR III, consistent with prior reports [7], (Figure 4B). However, expression profiles for the three NR4A-related genes that were elevated in the autoreactive CD4 T cell profiles shown in Figure 2 did not differ by INS genotype group in this high avidity-selected T cell population (Figure 5A).

## Discussion

The NR4A and EGR genes are prominent members of T cell immediate early response gene families [10], [11], [19], [20]. In murine studies with knockout strains, they have been found to play key roles in integrating TCR-initiated signals with other stimuli to determine cell fate decisions, most particularly in the thymus where they are sensors of TCR avidity [11]. In this manner, thresholds for T cell-positive and -negative selection are influenced by NR4A and EGR activity, integrated in the context of other stimuli, notably antigen density [17]. We report a direct relationship between proinsulin genotype and expression levels of NR4A and EGR genes in proinsulin-specific CD4 T cells, both in normal controls and in T1D subjects. A polymorphism in the proinsulin locus is associated with differential levels of proinsulin expression in the thymus, correlates with the frequency of circulating antigen-specific CD4 T cells in peripheral blood, and confers genetic susceptibility to T1D. After sorting these proinsulin-specific T cells based on tetramer-binding properties, we found higher levels of expression for NR4A1, NR4A2, and EGR3 in subjects with proINS VNTR I genotypes, the polymorphism associated with T1D risk. This relationship was most marked within a T cell population with intermediate binding avidity for the proinsulin-MHC tetramer.

Since tetramer-sorted T cells for these assays were expanded by antigen exposure prior to transcript analysis, the profiles observed represent a composite of the in vivo phenotype and the subsequent expression of the associated transcriptional program in vitro. Thus, the distinction in NR4A family profiles between the high and intermediate avidity cells may reflect programming during in vivo selection, changes in NR4A gene expression in high avidity cells during in vitro expansion, or both.

Autoreactivity is an intrinsic feature of the T cell repertoire, characterized by a spectrum of avidity for any particular peptide-MHC complex [21]–[23]. Human subjects with overt autoimmune disease tend to have a higher frequency of T cells for particular dominant autoantigen epitopes compared with control subjects, and these cells display memory markers, likely reflecting their experience of in vivo activation and expansion in the course of disease [13], [24]. While T cells with high avidity for self-antigens have a low threshold for both central and peripheral deletion, T cells with intermediate avidity for the same peptide-MHC target experience a threshold-dependent choice of fate, from anergy or regulation, to activation. This latter choice is incompletely understood, but appears to be programmed in part through the strength of signal mediated by the TCR-peptide-MHC interaction through pathways that involve signal modulators, including NR4A and EGR [10], [11]. In this manner, key functional decisions are programmed during early T cell maturation, which persist into the periphery, influencing response on subsequent contact with antigen.

We uncovered this type of programming mechanism linked to the differential expression of proinsulin in the thymus, associated with NR4A pathways in T cells that have intermediate avidity for this antigen. Thus, subjects with low levels of thymic proinsulin expression, genetically corresponding to VNTR I, not only fail to delete high avidity T cells, but also preferentially upregulate the NR4A and EGR gene families in the intermediate avidity T cells specific for proinsulin. In this manner, these genes act as translators of TCR signal strength, influencing cell fate. The discrimination observed between subjects with proinsulin VNTR I and VNTR III alleles indicates that avidity-dependent fate determination is under tight control, able to detect and respond to the differential level of TCR recognition associated with the proinsulin genetic polymorphism controlling antigen expression and disease susceptibility.

Only a few transcripts were observed to distinguish between T1D and control T cells specific for the same proinsulin-HLA complex, and these typically represent activation pathways likely to reflect the in vivo history of proinflammatory exposures in the course of disease. There was one notable interaction between INS genotype and T1D status, however, in which antigen-specific T cells from control subjects with the diabetes-protective proinsulin VNTR III preferentially expressed IL-10, IL-2, and FASLG, genes known to participate in potent regulatory pathways (Figure 5B). Since these increases in gene expression were observed only in the healthy, not in the T1D, subjects with the VNTR III genotype, these distinctions may be secondary to influences associated with protection from disease or with non-INS genetic differences. Interestingly, NR4A3 followed this pattern, and this was also the member of the NR4A family that showed asynchronous expression with NR4A1 and NR4A2 in the correlation with VNTR I seen in controls, but not in T1D subjects, as shown in Figure 2. Thus, it is possible that distinct members of the NR4A family have differential function in control subjects, with NR4A1 and NR4A2 associated with risk and NR4A3 with protection.

These studies support the concept that autoantigen-specific CD4 T cells represent a wide spectrum of TCR avidity and function. Translators of antigen signal strength, such as the NR4A and EGR gene families, represent key programming elements that likely participate in setting thresholds for particular functional outcomes, and thus have the potential to influence whether autoreactive T cells are anergic, pathogenic, or protective. It may be useful to monitor T cells after immunotherapy for autoimmune diseases to evaluate changes to these programming profiles, as one way to assess the durability of therapeutic response and the likelihood of disease recurrence.

## Materials and Methods

### Human subjects

Ethics statement. Adult patients diagnosed with T1D, according to the criteria of the American Diabetes Association, were recruited with informed consent as part of the Benaroya Research Institute and University of Rochester Institutional Review Boards approved studies, in writing, and samples were coded for non-identification.

A total of 68 HLA-DRB1*04:01-positive subjects was analyzed, of which seven were homozygous for DRB1*04:01; in addition, 18 had DRB1*03:01, and the remaining 43 had some other DRB1* allele. T1D patients of INS VNTR I/I, as well as INS VNTR I/III, genotype were of comparable age (average 27.3±7.5 years vs. 29.1±11.1 years, respectively), had similar duration of the disease (2.6±3.2 years vs. 4.1±2.8 years, respectively) and gender distribution (17 females and 9 males vs. 8 females and 6 males). Healthy control subjects (41.0±12.7 years vs. 49.3±13.2 years) of comparable gender distribution (10 females and 4 males vs. 9 females and 5 males) were similar. Seventeen subjects of both INS genotypes (ten healthy individuals and seven T1D patients) were evaluated in gene expression profiling analysis. Healthy individuals of both genotypes were of comparable age (37.3±9.8 years vs. 46.5±16.2 years), as were T1D patients (22.0±2.7 vs. 29.8±10.4), which had similar disease duration (4.6±2.8 years vs. 8.6±7.5 years).

### HLA typing and determination of INS VNTR genotype

Typing for HLA-DRB1* alleles and DRB1*04 subtypes was performed using sequence-specific primers and probes in conjunction with real-time PCR, as described [25]. The VNTR genotype was assigned based on the genotype at the -23 A/T single nucleotide polymorphism at the *INS* promoter [26]. Further subtyping for the polymorphisms within VNTR I alleles was not performed in this study [27].

### Tetramers

MHC class II tetramers were produced by constructing HLA class II expression vectors and generating soluble biotinylated HLA-DRB1*04:01 molecules, as described [28]. HLA molecules were loaded with peptides by detergent-facilitated exchange and multimerized by incubation with phycoerythrin-labeled streptavidin. Proinsulin 76–90 (SLQ**PLALEGSLQK**RG) [29], a 76–90 88S substituted peptide (SLQ**PLALEGSLQS**RG), where substitution of K88 to S in peptide position 9 enhanced its binding affinity and agonistic activity [14], and two irrelevant control peptides, an influenza A hemagglutinin, HA306-318 (KYVKQNTLKLA) [28] as a positive control and Borrelia burgdorferi OspA163-175 (KSYVLEGTLTAEK) as a negative control were each loaded into HLA-DRB1*04:01 molecules assembled into tetramers.

### Insulin-specific CD4+ T cell isolation

PBMC were isolated from heparinized blood and CD4+ T cells separated from non-CD4 cells using magnetic bead isolation (Miltenyi Biotec, Auburn, CA); CD25+ T cells were depleted using anti-CD25-coated microbeads. CD4+ CD25- T cells were cultured by restimulation with autologous adherent non-CD4+ cells as antigen-presenting cells, pre-pulsed with either native proinsulin 76–90 peptide or HA peptide (foreign antigen control), and without peptides (background control), and human recombinant IL-2 was added after day 6 of the culture. After 12–14 days of culture, T cells were stained with tetramers for flow cytometry analysis, as described [30].

### Gene expression profiling

Gene expression profiling was performed on tetramer-positive cells isolated by flow cytometry using a BD FACSVantage cell sorter. From the sorted tetramer-positive cells, total RNA was extracted using a MagMAX Total RNA Isolation Kit (Applied Biosystems, Inc., Foster City, CA). Alternatively, cells were lysed using Cells-to-Ct (Applied Biosystems, Inc.), and cDNA was prepared from the lysate without additional purification. First strand cDNA was synthesized with the RT^2^ First Strand Kit (SABiosciences, Corp., Frederick, MD), and samples were pre-amplified for a targeted assay panel using TaqMan Pre-amp Master Mix (Applied Biosystems, Inc.). Pre-amplified cDNA was combined with Gene Expression Assays (Applied Biosystems, Inc.) in the presence of Gene Expression Master Mix (Applied Biosystems, Inc.) and analyzed by real-time PCR on a StepOne Plus Sequence Detection System (Applied Biosystems, Inc.). Amplification was carried out in a total volume of 20 µl for 40 cycles of 15 sec at 95°C and 1 min at 60°C each. Each sample was amplified in duplicate. Due to the low numbers of proinsulin tetramer-positive cells in some subjects, not all subjects could be analyzed for a complete set of markers, thus resulting in different numbers of investigated individuals in some plots. Expression of each gene was determined by normalizing the expression to three housekeeping genes, and relative gene expression for each sample was calculated, as described by McDavid et al. [31]. Statistical comparisons of gene expression levels between INS-genotypes and between the groups of patients and controls were performed with a Mann-Whitney U test using GraphPad Prism 5 software.

## Supporting Information

Figure S1 **Relative expression profiles of flu-specific CD4+ T cells.** Profiles are shown in subjects of two INS genotypes matched for HLA-DRB1*04:01 and tested using the HA306-318 tetramer. Differences in the level of transcript expression between the groups were calculated using a Mann-Whitney U test. Horizontal lines indicate medians and inter-quartile ranges.
(PDF)

Table S1 **Panel of candidate genes used for transcription profiling.**
(TIF)
