# Supplementary figures and images for: Avidity-Dependent Programming of Autoreactive T Cells in T1D
Source: PLoS One. 2014 May 20;9(5):e98074. doi: 10.1371/journal.pone.0098074 (PMC4028311; doi:10.1371/journal.pone.0098074)

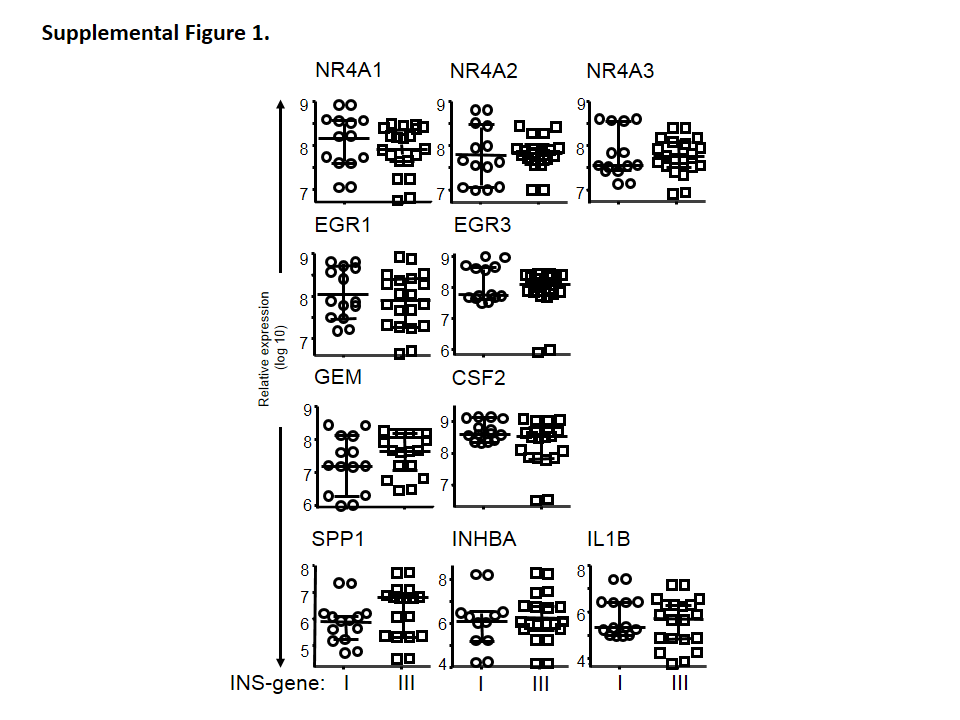

Supplement: Figure S1 — Relative expression profiles of flu-specific CD4+ T cells. Profiles are shown in subjects of two INS genotypes matched for HLA-DRB1*04:01 and tested using the HA306-318 tetramer. Differences in the level of transcript expression between the groups were calculated using a Mann-Whitney U test. Horizontal lines indicate medians and inter-quartile ranges. (PDF) [file pone.0098074.s001.pdf]
